# BteA Secreted from the *Bordetella bronchiseptica* Type III Secetion System Induces Necrosis through an Actin Cytoskeleton Signaling Pathway and Inhibits Phagocytosis by Macrophages

**DOI:** 10.1371/journal.pone.0148387

**Published:** 2016-02-01

**Authors:** Asaomi Kuwae, Fumitaka Momose, Kanna Nagamatsu, Yasuharu Suyama, Akio Abe

**Affiliations:** 1 Laboratory of Bacterial Infection, Graduate School of Infection Control Sciences, Kitasato University, Tokyo 108–8641, Japan; 2 Laboratory of Viral Infection II, Graduate School of Infection Control Sciences, Kitasato University, Tokyo 108–8641, Japan; University of Heidelberg Medical School, GERMANY

## Abstract

BteA is one of the effectors secreted from the *Bordetella bronchiseptica* type III secretion system. It has been reported that BteA induces necrosis in mammalian cells; however, the roles of BteA during the infection process are largely unknown. In order to investigate the BteA functions, morphological changes of the cells infected with the wild-type *B*. *bronchiseptica* were examined by time-lapse microscopy. L2 cells, a rat lung epithelial cell line, spread at 1.6 hours after *B*. *bronchiseptica* infection. Membrane ruffles were observed at peripheral parts of infected cells during the cell spreading. BteA-dependent cytotoxicity and cell detachment were inhibited by addition of cytochalasin D, an actin polymerization inhibitor. Domain analyses of BteA suggested that two separate amino acid regions, 200–312 and 400–658, were required for the necrosis induction. In order to examine the intra/intermolecular interactions of BteA, the amino- and the carboxyl-terminal moieties were purified as recombinant proteins from *Escherichia coli*. The amino-terminal moiety of BteA appeared to interact with the carboxyl-terminal moiety in the pull-down assay in vitro. When we measured the amounts of bacteria phagocytosed by J774A.1, a macrophage-like cell line, the phagocytosed amounts of *B*. *bronchiseptica* strains that deliver BteA into the host cell cytoplasm were significantly lower than those of strains that lost the ability to translocate BteA into the host cell cytoplasm. These results suggest that *B*. *bronchiseptica* induce necrosis by exploiting the actin polymerization signaling pathway and inhibit macrophage phagocytosis.

## Introduction

*Bordetella pertussis*, *B*. *parapertussis*, and *B*. *holmesii* are the causative agents of whooping cough (pertussis) [1, 2]. *B*. *bronchiseptica* infects several mammals, including rabbits, pigs, and dogs [3]. Most of the *Bordetella* virulence factors are regulated by the BvgAS two-component regulatory system at the transcriptional level. BvgS, which is a sensor histidine kinase localized in the inner membrane, is autophosphorylated in response to certain environmental signals. The phosphate group on the BvgS is transferred to BvgA, which is a transcriptional regulator of genes encoding many virulence factors [4]. The type III secretion system (T3SS) is also positively regulated by the BvgAS system [5].

A number of Gram-negative pathogenic bacteria produce a T3SS, which is a needle-like structure on outer surface of the bacterial body that acts as an injection nanomachine [6]. Upon infection of host cells, these bacteria also secrete translocase proteins, such as BopB [7] and BopD [8] in *Bordetella*, to form pores on the plasma membrane of host cells. Then, by utilizing the hollow needle of the T3SS and the newly formed membrane pores, the bacteria can inject proteins referred to as type III effectors directly into the host cell cytoplasm. In general, the injected type III effectors interact with host cell factors to disturb the host cell physiological functions. A recent report described that the T3SS of *B*. *pertussis* is activated in the mouse lung [9]. Another study showed that BopN is one of the type III effectors produced by *B*. *bronchiseptica* and contributes to bacterial colonization on the host respiratory tract by inducing IL-10 production and thereby suppressing inflammatory responses [10].

BteA, also referred to as BopC, is one of the type III effectors secreted from *B*. *bronchiseptica* [11, 12]. BteA was also shown to be secreted into the culture supernatant of the wild-type *B*. *bronchiseptica*, but not into the culture supernatant of a T3SS-deficient mutant [12]. BteA appeared to be translocated into the host cell cytoplasm through its amino (N)-terminal 48 amino acid region. *B*. *bronchiseptica* induces morphological changes, dephosphorylation of tyrosine-phosphorylated proteins, and necrosis of cultured mammalian cells in a BteA-dependent manner [12]. Finally, it was reported that exogenous expression of BteA in cultured mammalian cells by eukaryotic expression vector induces necrosis, and that BteA is localized on the lipid raft domains of the host cell plasma membrane through its N-terminal region [13].

Nevertheless, although BteA is known to be involved in the host responses described above, the molecular mechanisms underlying these phenomena are largely unknown. In this study, we investigated the precise mechanisms of the BteA-mediated necrosis and the significance of BteA functions for *Bordetella* infection.

## Materials and Methods

### Bacterial strains and cell culture

*B*. *bronchiseptica* S798 was used as the wild-type strain [7]. The other *B*. *bronchiseptica* strains, i.e., ΔBteA, ΔBteA/pBteA, ΔBopN, ΔBspR, and ΔBscN, which was used as the type III secretion system-deficient strain (ΔT3SS), were described previously [7, 10, 12, 14]. *B*. *bronchiseptica* was grown on a Bordet-Gengou agar plate at 37°C for 48 hours. *B*. *bronchiseptica* from fresh colonies on the Bordet-Gengou agar plates were suspended in Steiner-Sholte liquid (SS) medium [15] composed of 1 L of basal medium (containing 11.84 g of mono-sodium glutamate monohydrate, 0.24 g of L-proline, 2.50 g of NaCI, 0.50 g of KH_2_PO_4_, 0.20 g of KCI, 0.10 g of MgCl_2_•6H_2_O, 0.02 g of CaCl_2_, 6.1 g of Tris, 10 g of casamino acids, and 1 g of heptakis(2, 6-di-*O*-methyl)-β-cyclodextrin) and 10 mL of supplement (containing 40 mg of L-cysteine hydrochloride, 10 mg of iron(II) sulfate heptahydrate, 400 mg of L-ascorbic acid, 4 mg of nicotinic acid, and 150 mg of reduced L-glutathione). A starting OD_600_ was adjusted to 0.2 when *B*. *bronchiseptica* was grown in SS medium. *Escherichia coli* DH10B (Invitrogen), KRX (Promega), and BL21(DE3) (Novagen) were used for cloning, HaloTag protein production, and six histidine residues (6 x His)-tagged protein production, respectively.

A rat lung epithelial cell line, L2 cells (ATCC CCL-149), An African monkey kidney fibroblast-like cell line, COS-7 cells (ATCC CRL-1651), and a mouse macrophage-like cell line, J774A.1 cells (ATCC TIB-67) were grown in F-12K, DMEM, and RPMI medium, respectively. A mouse dendritic cell line DC2.4 was provided by K. M. Rock (University of Massachusetts, Worcester, MA) and grown in a cell culture medium RPMI containing 55 μM 2-mercaptoethanol. Each cell culture medium contained 10% of fetal bovine serum (FBS). Cultured mammalian cells were grown at 37°C under a 5% CO_2_ atmosphere.

### Plasmid construction

The oligonucleotides used in this study are listed on Table 1. In order to construct plasmids for gene expression in mammalian cells, we amplified DNA fragments encoding the full length (amino acid region 1–658), N-terminal moiety (amino acid region 1–312), or carboxyl (C)-terminal moiety (amino acid region 313–658) of BteA with the primer sets of 5-bteA and 3-bteA, 5-bteA and 3-bteA-312, or 5-bteA-313 and 3-bteA, respectively, using *B*. *bronchiseptica* S798 genomic DNA as a template. Each amplified DNA fragment was digested with BglII and HindIII and then cloned into the BamHI and HindIII recognition sites of pcDNA3.1-myc-His(A) (Invitrogen). The resulting plasmids were designated as pcDNA-BteA-FL, pcDNA-BteA-N312, and pcDNA-BteA-C313, respectively. In order to obtain a plasmid encoding amino acid regions 1–600, 1–510, 1–490, 1–400, 1–200, 200–658, 400–658, or 500–658 of BteA, we amplified the DNA fragment with the primer sets of pcDNA-HindIII-U and bteA-N600, pcDNA-HindIII-U and bteA-N510, pcDNA-HindIII-U and bteA-N490, pcDNA-HindIII-U and bteA-N400, pcDNA-HindIII-U and bteA-N200, bteA-C200 and pcDNA-Koz-L, bteA-C400 and pcDNA-Koz-L, or bteA-C500 and pcDNA-Koz-L using pcDNA-BteA-FL as a template. The amplified fragment was self-ligated by an In-Fusion Cloning System (Clontech). The resulting plasmids were designated as pcDNA-BteA-N600, pcDNA-BteA-N510, pcDNA-BteA-N490, pcDNA-BteA-N400, pcDNA-BteA-N200, pcDNA-BteA-C200, pcDNA-BteA-C400, and pcDNA-BteA-C500, respectively. In order to obtain a plasmid encoding amino acid region 200–400 of BteA, we amplified the DNA fragment with the primer set of pcDNA-HindIII-U and bteA-N400 using pcDNA-BteA-C200 as a template. The amplified fragment was self-ligated and the resulting plasmid was designated as pcDNA-BteA-N2-4.

**Table 1:** Oligonucleotides used in this study.

Name	Sequence
5-bteA	GAA GAT CTC GCC ACC ATG GTG AGC AAC AAC GTC AAT CCG GTC
3-bteA	CCC AAG CTT TGC GCG TAG ATT CAG CGC
3-bteA-312	CCC AAG CTT GGC CTT GGC ACC GGC TTC GAC GG
5-bteA-313	GAA GAT CTC GCC ACC ATG GGC ACC GAT TTC GAG GCG CCG
pcDNA-HindIII-U	AAG CTT GGG CCC GAA CAA AAA CTC ATC
bteA-N600	TTC GGG CCC AAG CTT TTG GCT GGT CGC GTC GAT GTG
bteA-N510	TTC GGG CCC AAG CTT GGC CGT GGC TCC CAG TCC GAC
bteA-N490	TTC GGG CCC AAG CTT GTC GGC ATA TTT GCT GTG CAG
bteA-N400	TTC GGG CCC AAG CTT GGC GCT GGC CGC GGT GTC GC
bteA-N200	TTC GGG CCC AAG CTT CAT GCC CGC GAC GTC GCT CG
bteA-C200	CTC GCC ACC ATG GTG ATG CCG CAA TGG CAG GAA TAC
bteA-C400	CTC GCC ACC ATG GTG GCC AAC CCC GTG CTG TCG CTG
bteA-C500	CTC GCC ACC ATG GTG GGT AGC GCC AAT GTC GGA CTG
pcDNA-Koz-L	CAC CAT GGT GGC GAG ATC CAC TAG
5-HindIII-bteA	ATC CGA ATT CAA GCT TTT GAG CAA CAA CGT CAA TCC GGT CG
3-Strep-bteA	CGG GTG GCT CCA TGC GCG TAG ATT CAG CGC CG
3-Strep-bteA-312	CGG GTG GCT CCA GGC CTT GGC ACC GGC TTC GAC GG
5-HindIII-bteA-313	ATC CGA ATT CAA GCT TAG CAC CGA TTT CGA GGC GCC GGC CAG C
3-HindIII-Strep	GCA GGT CGA CAA GCT TTC ATT TTT CGA ACT GCG GGT GGC TCC A
pCold-FLAG-U	CAA GGA TGA CGA TGA CAA GTG AAA GCT TGT CGA CCT GCA G
pCold-FLAG-L	TCA TCG TCA TCC TTG TAA TCT GCG CGT AGA TTC AGC GCC G
5-SgfI-bteA	CAG AGC GAT AAC GCG ATC GCG TTG AGC AAC AAC GTC AAT CCG GTC
3-FLAG-bteA-312	ATC CTT GTA ATC GGC CTT GGC ACC GGC TTC GAC GG
5-SgfI-btcA	CAG AGC GAT AAC GCG ATC GCG TTC CCT TTG CTC ATC CGC AAT CTG
3-FLAG-btcA	ATC CTT GTA ATC GGC CTT GCG GCC GGC GAC CTC G
3-PmeI-FLAG	AGC CCG AAT TCG TTT AAA CTC ACT TGT CAT CGT CAT CCT TGT AAT C

Restriction enzyme recognition sites were underlined.

In order to purify BteA tagged with 6 x His at the N-terminal and with Strep tag at the C-terminal, the following plasmids were constructed. We amplified the DNA fragment encoding the full length, the N-terminal moiety (amino acid region 1–312), or the C-terminal moiety (amino acid region 313–658) of BteA with the primer set of 5-HindIII-bteA and 3-Strep-bteA, 5-HindIII-bteA and 3-Strep-bteA-312, or 5-HindIII-bteA-313 and 3-Strep-bteA, respectively, using *B*. *bronchiseptica* S798 genomic DNA as a template. Each amplified DNA fragment was used as a template for the 2^nd^ PCR with a primer set consisting of the upper primer used in the 1^st^ PCR and 3-HindIII-Strep to add a 24 bp sequence encoding the Strep tag. The resulting amplified DNA fragments were cloned into the HindIII recognition site of pColdII (TAKARA) by an In-Fusion Cloning System, yielding pCold-bteA-Strep encoding the full length of BteA, pCold-bteA-N-Strep encoding amino acid region 1–312 of BteA, and pCold-bteA-C-Strep encoding amino acid region 313–658 of BteA. In order to purify BteA and BtcA tagged with 6 x His at the N-terminal and with FLAG tag at the C-terminal, the following plasmids were constructed. We performed PCR reactions with the FLAG tag sequence-containing primer set of pCold-FLAG-U and pCold-FLAG-L using pCold-bteA-Strep or pCold-bteA-C-Strep as a template to replace the sequence encoding the Strep tag with the sequence encoding FLAG tag. The resulting PCR products were self-ligated by an In-Fusion Cloning System, and pCold-bteA-FLAG encoding the full length of BteA and pCold-bteA-C-FLAG encoding amino acid region 313–658 of BteA were obtained. We also amplified the DNA fragment encoding the N-terminal moiety (amino acid region 1–312) of BteA or the full length of BtcA with the primer set of 5-SgfI-bteA and 3-FLAG-bteA-312 or 5-SgfI-btcA and 3-Strep-btcA, respectively, using *B*. *bronchiseptica* S798 genomic DNA as a template. Each amplified DNA fragment was used as a template for the 2^nd^ PCR with a primer set consisting of the upper primer used in the 1^st^ PCR and 3-PmeI-FLAG to add a 24 bp sequence encoding the FLAG tag. Each of the resulting amplified DNA fragments was cloned into the SgfI and PmeI recognition sites of pFN18A (Promega) by using an In-Fusion Cloning System, yielding pFN-bteA-N-FLAG encoding amino acid region 1–312 of BteA and pFN-btcA-FLAG encoding the full length of BtcA. Both the N-terminal moiety of BteA and full length of BtcA produced in *E*. *coli* KRX (Promega) harboring each plasmid were tagged with HaloTag at the N-terminal and with FLAG tag at the C-terminal.

### Transfection assay of cultured mammalian cells

Introduction of plasmids into cultured mammalian cells was performed with Lipofectamine LTX (Invitrogen) according to the manufacturer’s instruction.

### Live imaging of L2 cells infected with *B*. *bronchiseptica*

In order to analyze the morphological changes of cultured mammalian cells infected with bacteria, 1 x 10^5^ cells of L2 or 3 x 10^5^ cells of L2 were grown in 35 mm glass-bottomed dishes overnight and pEGFP-C1 (Clontech) was then introduced into the cells. An overnight culture of wild-type *B*. *bronchiseptica* in SS medium was added into the extracellular medium of the transfected cells at a multiplicity of infection (MOI) of 1000 without centrifugation. Live-cell imaging was performed by a fluorescence microscope (IX71, Olympus Optical) equipped with a stage-top incubation chamber, a microlens-enhanced Nipkow-disk confocal scanner unit (CSU-X1, Yokogawa Electric), and an electron-multiplying CCD camera (Luca, Andor Technology). The microscope and camera were controlled by Andor iQ 1.8 software (Andor Technology). Acquired images were processed by ImageJ 1.43u software. [16]. Infected cells were incubated at 37°C under a 5% CO_2_ atmosphere.

### Measurement of LDH amount released from L2 cells infected with *B*. *bronchiseptica*

In order to analyze whether cytochalasin D and latrunculin B affects the cytotoxicity of *B*. *bronchiseptica*, 3 x 10^4^ cells/well of L2 were grown in 24-well plates overnight for measurement of the amounts of lactate dehydrogenase (LDH) released into extracellular medium by a Cytotox 96 Non-Radioactive Cytotoxicity Assay kit (Promega). The cells were treated with cytochalasin D (Sigma-Aldrich), latrunculin B (Calbiochem), or MG-132 (Sigma-Aldrich) at 5 μM, 1 μM, or 10 μM, respectively, for 1 hour. When inhibitors were removed from the media, the cells were washed twice with cell culture media. An overnight culture of *B*. *bronchiseptica* in SS medium was then added to the extracellular medium at an MOI of 100. The plates were centrifuged at 900 x *g* for 5 minutes and incubated for 1 hour at 37°C under a 5% CO_2_ atmosphere, and the extracellular media were then recovered for measurement of the LDH amounts. We measure the LDH amount in the medium of uninfected cells control and use the value as background. The absorbance value obtained from uninfected cells was subtracted from all of the absorbance values obtained from cells infected with bacteria or treated by Triton X-100. The LDH (%) was shown as a ratio when the value obtained from the well treated with 0.1% Triton X-100 was set as 100%.

### Immunofluorescence staining of L2 cells infected with *B*. *bronchiseptica*

In order to analyze whether cytochalasin D and latrunculin B affects the cell morphologies of L2 cells infected with *B*. *bronchiseptica*, 1 x 10^5^ cells/well of L2 were grown in 6-well plates for immunofluorescence staining overnight. The cells were treated with inhibitors as described above. An overnight culture of *B*. *bronchiseptica* in SS medium was then added to the extracellular medium at an MOI of 100. The plates were centrifuged at 900 x *g* for 5 minutes and incubated for 1 hour at 37°C under a 5% CO_2_ atmosphere, and the cells were fixed in 4% paraformaldehyde for immunofluorescence staining. *B*. *bronchiseptica* were labeled with anti-*B*. *bronchiseptica* antisera (Denkaseiken) and Alexa 488 –labeled anti-rabbit IgG (Invitrogen). F-actin was stained with rhodamine phalloidin (Roche).

### LDH release assay and lysate preparation after plasmid introduction

In order to examine whether the proteins exogenously produced in mammalian cells by transfection of each pcDNA3.1-myc-His(A)-derived plasmid induce necrosis, COS-7 cells were seeded in 24-well plate at 7.5 x 10^4^ cells/well and grown overnight. The cells were transfected and the amounts of LDH released into the extracellular medium were measured at 24 hours after transfection. We measured the LDH amount in the medium of uninfected cells as a control and use the value as background. The absorbance value obtained from untransfected cells was subtracted from all of the absorbance values obtained from cells transfected with each plasmid or treated by Triton X-100. The LDH (%) was shown as a ratio when the value obtained from the well treated with 0.1% Triton X-100 was set as 100%. In order to examine whether the proteins were exogenously produced in mammalian cells, the transfected cells were also washed with PBS twice and lysed in sodium dodecyl sulfate-polyacrylamide gel electrophoresis (SDS-PAGE) sample buffer. The samples were incubated at 95°C for 5 minutes and then subjected to SDS-PAGE with 12% gel. The proteins in the gel were transferred to a polyvinylidene difluoride (PVDF) membrane and the Myc-tagged proteins were detected by Western blot with anti-Myc 9E10 antibodies (Santa Cruz).

### Purification of BteA and pull-down assay

We purified 6 x His-tagged proteins and HaloTag fusion proteins using Ni-NTA agarose (Qiagen) and HaloLink resin (Promega), respectively, according to the manufacturer’s instructions. HaloTag fusion proteins were digested by ProTEV Protease to remove HaloTag. The purified proteins were dialyzed with PBS. We mixed 1.0 μg of the N-terminal moiety of BteA (38.0 kDa) or 1.0 μg of the C-terminal moiety of BteA (38.5 kDa) tagged with Strep tag at the respective C-terminus with 1.9 μg of the full length of BteA (72.6 kDa), 0.9 μg of the N-terminal moiety of BteA (35.7 kDa) or 1.0 μg of the C-terminal moiety of BteA (38.5 kDa), or 0.4 μg of BtcA (15.4 kDa) tagged with FLAG at the respective C-terminus. We mixed two kinds of purified proteins at the same molecular numbers as described above in 50 μl of PBS containing 1% Triton X-100 and incubated the mixture at room temperature for 1 hour. We added 25 μl of Strep-Tactin resin (IBA) to each tube and rotated the tubes at room temperature for an additional 1 hour. We then transferred 15 μl of supernatant into new eppendorf tubes and add 15 μl of 2 x SDS-PAGE sample buffer to prepare supernatant fractions. The resulting Strep-Tactin resins were washed with PBS containing 1% Triton X-100 and 30 μl of SDS-PAGE sample buffer was then added to each washed resin to prepare precipitated fraction. The samples were incubated at 95°C for 5 minutes. The 12.5% of total input of supernatant or precipitated fractions (5 μl/lane) were then subjected to SDS-PAGE with 12% gel. FLAG-tagged or Strep-tagged proteins were detected in Western blot with anti-FLAG M2 antibody (Sigma-Aldrich F3165) or anti-Strep antibody (NWSHPQFEK antibody, rabbit polyclonal antibody, GenScript, A00626-40), respectively.

### Gentamicin protection assay

The gentamicin protection assay was performed as described previously [17]. Briefly, J774A.1 cells were grown in 24-well plates at 7.5 x 10^4^ cells/well overnight. A *B*. *bronchiseptica* overnight culture in SS medium was then added to the extracellular medium of the J774A.1 cells at an MOI of 100 without centrifugation. Gentamicin was added at a final concentration of 1 mg/ml at 1 hour post-infection. After 30 minutes of gentamicin treatment in a CO_2_ incubator, phagocytosed bacteria were recovered with phosphate-buffered saline (PBS) containing 0.5% Triton X-100 and the resulting bacterial suspension was plated on an LB plate to count the number of colonies.

### Immunofluorescence of DC2.4 cells infected with *B*. *bronchiseptica*

In order to examine bacterial localizations during infection of phagocytes, 4 x 10^5^ cells/well of DC2.4 cells were grown in 6-well plates. An overnight culture of *B*. *bronchiseptica* in SS medium was then added to the extracellular medium at an MOI of 100. The plates were centrifuged at 900 x *g* for 5 minutes and incubated for 30 minutes at 37°C under a 5% CO_2_ atmosphere, and the cells were fixed in 4% paraformaldehyde for immunofluorescence staining. *B*. *bronchiseptica* and F-actin were stained as described above. The images were captured by Axioplan 2 (Zeiss) and the deconvolution analyses were performed in Imaris 5.0.3 software (Bitplane).

### Statistics

Statistical analyses were performed using nonparametric unpaired t-test of one tailed *p*-value on Prism 5.0f software (Graphpad). The values of *p*<0.05 is considered statistically significant.

## Results

### Morphological changes of cells infected with the wild-type *B*. *bronchiseptica*

**Fig 1:**
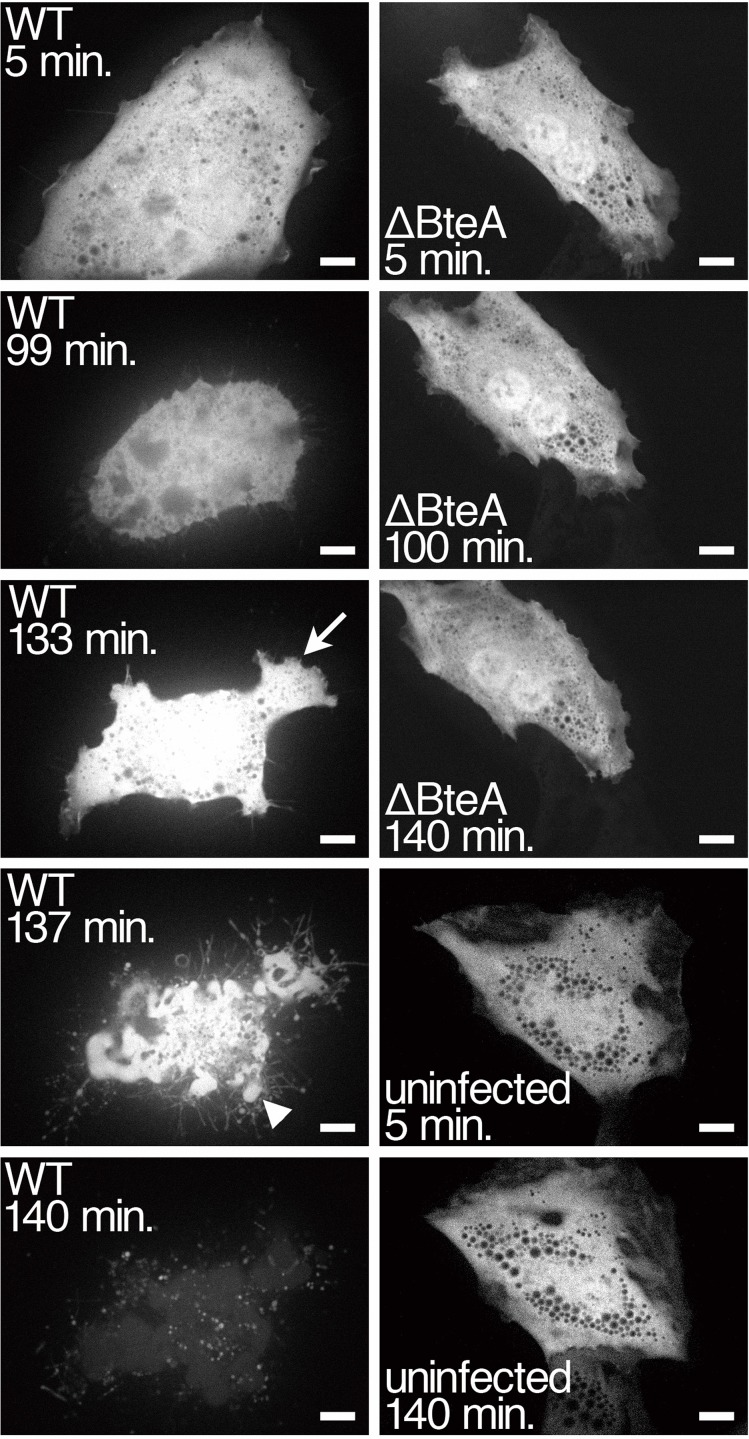
Time-lapse analyses of morphological changes of *B*. *bronchiseptica*-infected mammalian cells. The pEGFP-C1 plasmid encoding EGFP was introduced into L2 cells to look at the morphologies of the cells under fluorescent microscope. The transfected L2 cells were infected with *B*. *bronchiseptica* strains in a glass-bottomed dish. The infected cells were analyzed under a confocal laser scanning microscope equipped with a time-lapse CCD camera. A representative cell of each type at the indicated time post-infection is shown. Arrows and arrowheads indicate membrane ruffles and blebs, respectively. Scale bar shows 10 μm. Data are representative of three independent experiments.

It has been shown that *B*. *bronchiseptica* infection induces morphological changes such as detachment and shrinking in cultured mammalian cells [7, 12]. In order to further analyze the processes of the *Bordetella*-induced morphological changes, time-lapse live images of infected cells were captured. In order to simulate natural infection, we used a rat lung epithelial cell line, L2, for the infection assays. The pEGFP-C1 plasmid encoding EGFP was introduced into L2 cells to look at the morphologies of the cells under fluorescent microscope. The transfected L2 cells were infected with *B*. *bronchiseptica* strains in a glass-bottomed dish. The images of infected cells were captured by a confocal laser scanning microscope equipped with a CO_2_ chamber. At 99 minutes after infection, the L2 cells began to shrink in the representative sample (Fig 1 and S1 Movie). At 133 minutes after infection, the L2 cells spread with membrane ruffles at the cell periphery (Fig 1, arrow). After spreading, the L2 cells collapsed with membrane blebs (Fig 1, arrowhead) and then detached from the substrata. In contrast, L2 cells infected with the BteA-deficient strain (ΔBteA) or uninfected L2 cells did not induce the morphological changes even at 150 minutes post-infection (Fig 1, S2 and S3 Movies).

**Fig 2:**
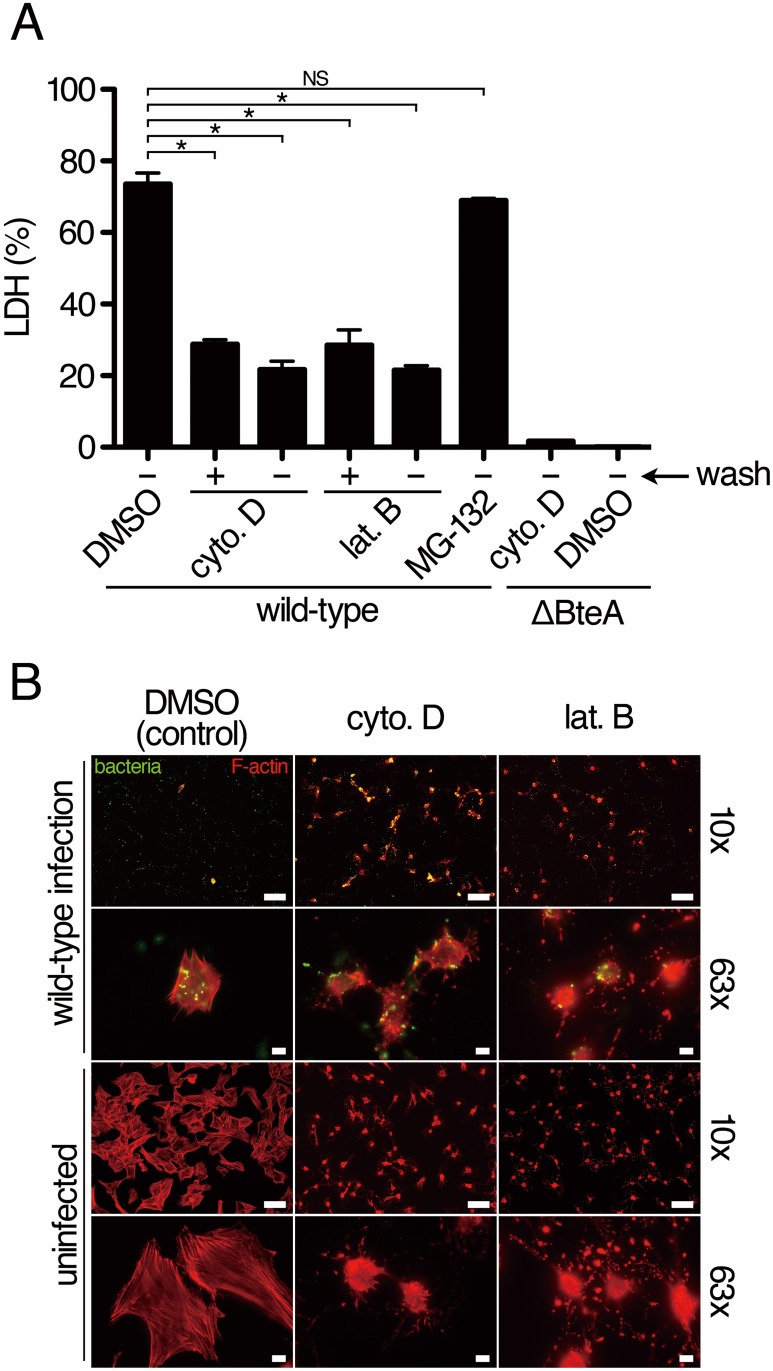
*B*. *bronchiseptica* infection with cytochalasin D treatment. A. L2 cells were treated with cytochalasin D (cyto. D), latrunculin B (lat. B), MG-132, or DMSO for 1 hour. The inhibitors were washed (+) or not washed (-) with the cell culture media. The cells then infected with the wild-type or BteA-deficient strain (ΔBteA) of *B*. *bronchiseptica*. At 1 hour post-infection, the amount of LDH released into the extracellular medium was measured. The error bars represent the standard error of the mean (SEM) from triplicate experiments. An asterisk (*) and NS show a significant difference (*p* < 0.05) and no significant difference, respectively. Representative data from one of three independent experiments were shown. B. Actin polymerization inhibitor-treated L2 cells or DMSO-treated cells (control) were fixed after 1 hour of infection with the wild-type *B*. *bronchiseptica*. The infected bacteria (green) and F-actin (red) were stained with anti-*B*. *bronchiseptica* antisera and rhodamine phalloidin, respectively. The objectives of 10x (numerical aperture: 0.30) and 63x (numerical aperture: 1.40) were used to acquire the images. The upper six photos show the cells infected with the wild-type *B*. *bronchiseptica* and the lower six photos show uninfected cells. Scale bars in photos obtained with 10x or 63x objective show 100 or 10μm, respectively. Representative data from one of three independent experiments.

Upon formation of membrane ruffles, actin polymerization is generally required [18]. In order to investigate the relationship between necrosis and membrane ruffles during *B*. *bronchiseptica* infection, L2 cells were treated with actin polymerization inhibitors, such as cytochalasin D and latrunculin B, and proteasome inhibitor, for 1 hour. The inhibitor-treated L2 cells were then infected with the wild-type *B*. *bronchiseptica* or ΔBteA for 1 hour, and the degree of necrosis was measured by lactate dehydrogenase (LDH) release as described in the Materials and Methods. The amount of LDH released from cells infected with the wild-type strain was significantly reduced by the actin polymerization inhibitor treatment, even inhibitors were washed out before infection (Fig 2A), while no significant difference of LDH release was obtained from the cells treated with MG-132, a proteasome inhibitor, used as a control. Even when we washed actin polymerization inhibitors before infection, the LDH release was significantly reduced (Fig 2A). We also confirmed that *Bordetella*-induced necrosis was dependent on the BteA function (Fig 2A). Next, L2 cells were treated with cytochalasin D or latrunculin B for 1 hour and then, without washing, infected with the wild-type *B*. *bronchiseptica* for 20 minutes. After infection, the F-actin and infected bacteria were stained. As reported previously [12], most of the cells that were not treated with actin polymerization inhibitors were detached from the substrata by *B*. *bronchiseptica* infection, while the cells treated with cytochalasin D or latrunculin B were still attached to substrata (Fig 2B). These results suggest that the actin polymerization is required for the necrosis and cell detachment induced by *B*. *bronchiseptica* infection.

### LDH release induced by truncated versions of BteA

**Fig 3:**
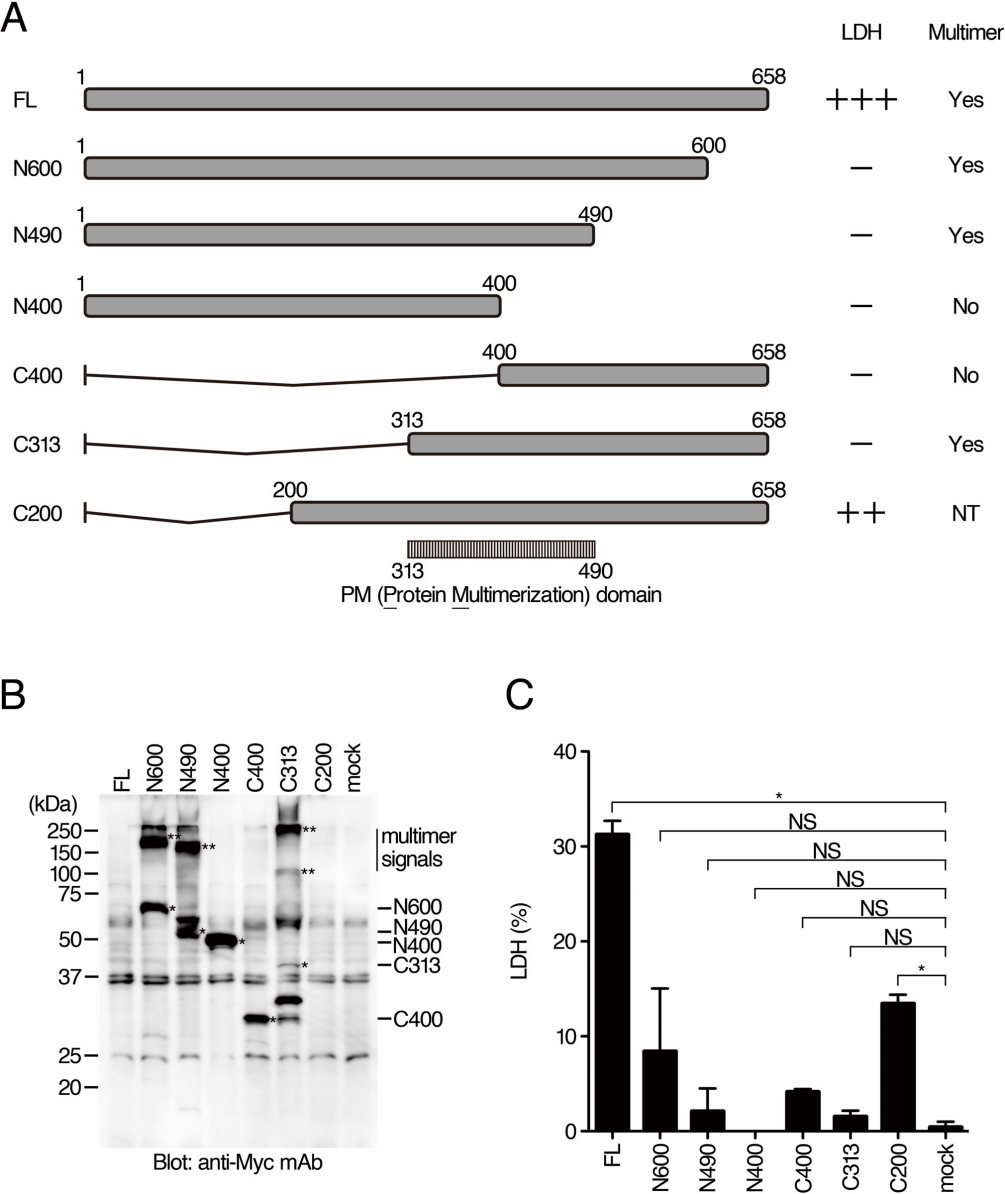
BteA multimerization through amino acids region 313–490. A. Truncated versions of BteA encoded by the plasmids used for transfection are depicted. Results of LDH release assays (C) and multimer detection status (B) are shown on the right side of the panel. The released LDH levels were shown as 0 (-), 30–70 (++), or more than 70% (+++) of the amount of LDH released from cells that the plasmid encoding full length is introduced. NT: not tested. B. The results of Western blot analysis with anti-Myc antibodies are shown. The subjected samples were lysates recovered from cells transfected with the plasmids encoding the respective truncated versions of BteA. Monomer and multimer signals are indicated as * and **, respectively. C. The results of LDH release assays are shown as a histogram. The error bars represent SEM from triplicate experiments. All conditions were compared with mock. Asterisk (*) and NS show a significant difference (*p* < 0.05) and no significant difference, respectively. Representative data from one of three independent experiments were shown.

It has been shown that exogenous expression of BteA in cultured mammalian cells by eukaryotic expression vector induces necrosis [11, 12]. In order to determine the BteA regions responsible for necrosis induction, various DNA fragments encoding truncated versions of BteA were inserted into a eukaryotic expression vector containing SV40 replication origin. We used COS-7 cells for transfection because COS-7 cells produce the SV40 large T antigen to enhance the replication of plasmid containing the SV40 replication origin. Therefore, we are able to expect high expression levels of target genes. At 24 hours after introduction of the plasmids into COS-7 cells, the amounts of LDH released into the extracellular media were measured. As shown in Fig 3, LDH release was detected by the introduction of pcDNA-BteA-C200 (encoding amino acid region 200–658 of BteA) or pcDNA-BteA-FL (encoding the full length of BteA). The level of LDH release by introduction of pcDNA-BteA-C200 was about half of that of pcDNA-BteA-FL. On the other hand, LDH release was not detected by introduction of other plasmids. These results suggest that the N-terminal 199 amino acid region is not essential for necrosis induction. As reported previously, BteA forms a multimer in the culture supernatant fraction and bacterial lysate [12]. COS-7 cell lysates were also prepared from samples used for the LDH release assay, and BteA was detected by Western blot analysis with anti-Myc antibodies. In the cell lysate, multimer signals corresponding to amino acid region 1–600 (N600), 1–490 (N490), and 313–658 (C313) of BteA were detected (Fig 3B). The cells producing the full length or C200 of BteA are disrupted and the BteA proteins are probably released into the culture medium. Therefore, we did not detected signals for the full length and C200 of BteA in the western blot analysis. These results suggest that the region responsible for multimerization (PM domain, Fig 3A) is included in amino acid region 313–490 of BteA.

### Necrosis induction by two separate domains of BteA produced in trans

**Fig 4:**
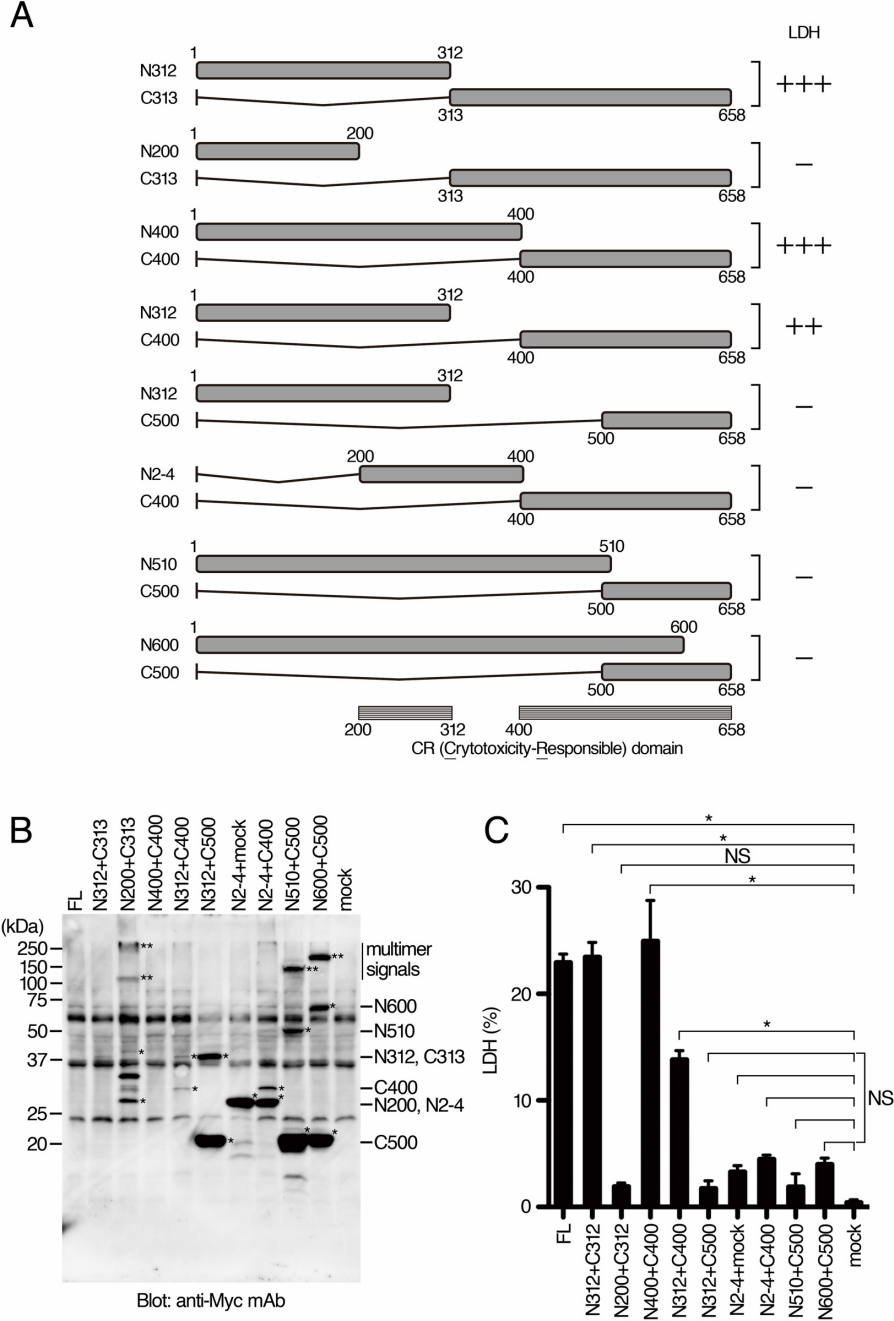
Two separate domains of BteA induce LDH release. A. Two kinds of plasmids were simultaneously introduced into COS-7 cells in order to produce BteA regions as individual polypeptides. The pairs of two truncated versions simultaneously introduced are depicted. Results of LDH release assays (C) are shown on the right. The released LDH levels were shown as 0 (-), 30–70 (++), or more than 70% (+++) of the amount of LDH released from cells that the plasmid encoding full length is introduced. B. The results of Western blot analysis with anti-Myc antibodies are shown. Monomer and multimer signals are indicated as * and **, respectively. C. The results of LDH release assays are shown as a histogram. The error bars represent SEM from triplicate experiments. All conditions were compared with mock. Asterisk (*) and NS show a significant difference (*p* < 0.05) and no significant difference, respectively. Representative data from one of three independent experiments.

To examine the possibility that the domain responsible for the necrosis induction is separated within BteA molecule, pcDNA-BteA-N312 (encoding amino acid region 1–312 of BteA) and pcDNA-BteA-C313 (encoding amino acid region 313–658 of BteA) were simultaneously introduced into COS-7 cells. The LDH release was not detected in cells transfected with either pcDNA-BteA-N312 or pcDNA-BteA-C313 alone (data not shown, Fig 3). In contrast, the level of LDH released from cells into which both pcDNA-BteA-N312 and pcDNA-BteA-C313 were introduced was similar to that from the cells transfected with pcDNA-BteA-FL (encoding the full length of BteA) alone (Fig 4). The LDH release was also detected by the introduction of plasmid pairs of pcDNA-BteA-N400 (encoding amino acid region 1–400 of BteA) + pcDNA-BteA-C400 (encoding amino acid region 400–658 of BteA) or pcDNA-BteA-N312 + pcDNA-BteA-C400 into COS-7 cells (Fig 4). However, the LDH release was not detected by the introduction of other plasmid pairs. These results indicate that the two separate domains of amino acid region 200–312 and 400–658 of BteA (CR domain, Fig 4A) are essential for the necrosis induction even if these regions are produced as individual polypeptides.

### BteA C-terminal moiety interacts with BteA N-terminal and the C-terminal moiety itself

**Fig 5:**
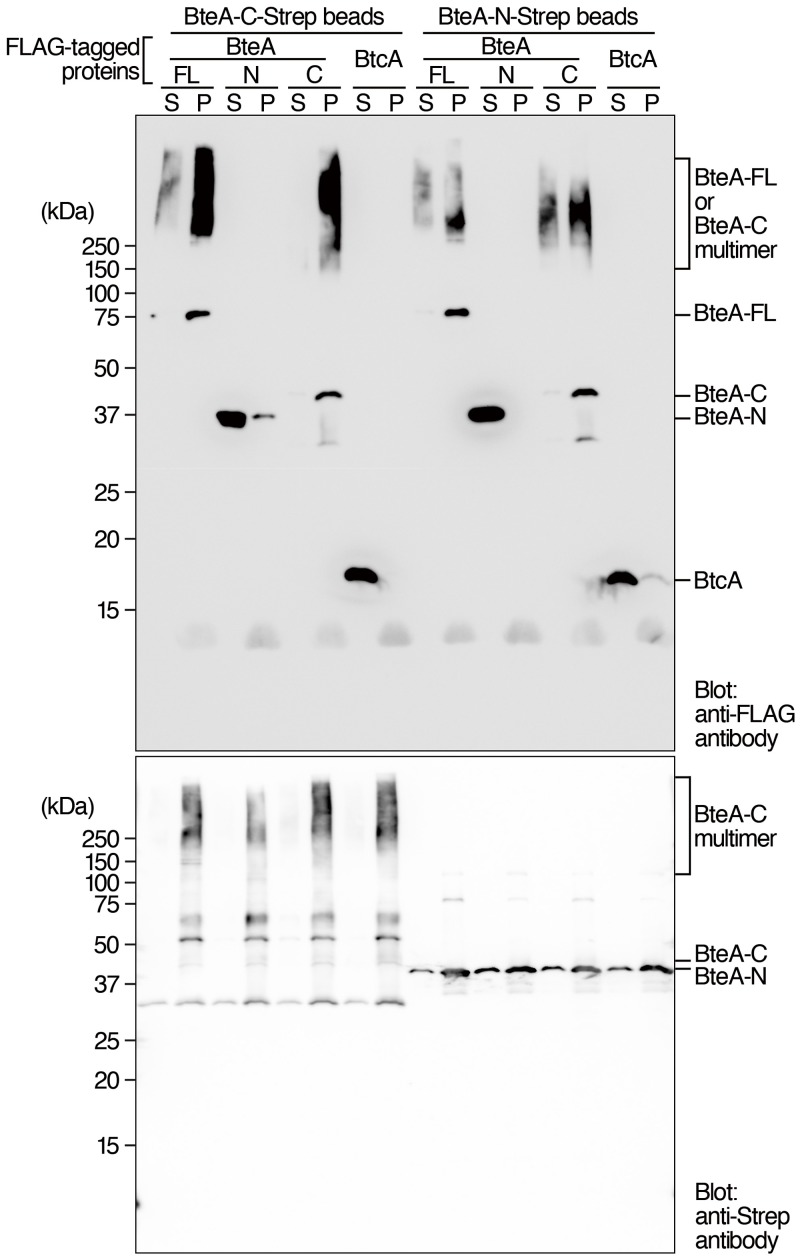
BteA C-terminal moiety interacts with BteA N-terminal and the C-terminal moiety itself. The recombinant full length (FL), N-terminal moiety (amino acid region 1–312; N), and C-terminal moiety (amino acid region 313–658; C) of BteA, and BtcA were purified as FLAG- or Strep-tagged protein. Each FLAG-tagged protein was mixed with Strep-tagged proteins. Streptactin beads were added to the tubes in order to pull-down Strep-tagged proteins and interacting FLAG-tagged proteins. After 1 hour incubation, the supernatant was transferred to new eppendolf tubes to prepare supernatant fractions (S). The resulting streptactin beads were washed and used for preparation of precipitated fractions (P). One sixth (12.5%) of total input was loaded to each lane of 12.5% of SDS-PAGE gel. The upper panel and lower panel show the results of Western blot with anti-FLAG antibodies (Sigma-Aldrich F3165) and anti-Strep antibodies (GenScript A00626-40), respectively. Data are representative of three independent experiments.

To examine whether the N-terminal moiety of BteA interacts with the C-terminal moiety of BteA, pull-down assays were carried out. We purified the full-length BteA (amino acid region 1–658), the N-terminal moiety of BteA (amino acid region 1–312), and the C-terminal moiety of BteA (amino acid region 313–658) as recombinant proteins produced by *E*. *coli*. We also purified recombinant BtcA protein that interacts with the N-terminal region of BteA in vitro [11] in order to use it as a positive control for interaction with BteA. These purified proteins were tagged with a FLAG or Strep sequence. We mixed the Strep-tagged protein and FLAG-tagged protein at room temperature for 1 hour, then added streptactin resin to precipitate the Strep-tagged protein. After additional 1 hour incubation, supernatant (S) and precipitated (P) fractions were prepared. The resulting S and P fractions were separated by SDS-PAGE and analyzed by Western blot with anti-FLAG antibodies. Signals of the full length, the N-terminal moiety, and the C-terminal moiety, but not BtcA were detected in the protein sample precipitated by the Strep-tagged C-terminal moiety of BteA (Fig 5). Signals of the full length, and C-terminal moiety, but not the N-terminal moiety were detected in the protein sample precipitated by the Strep-tagged N-terminal moiety of BteA (Fig 5). Although the BtcA was detected as faint signal, BtcA was pulled down by the N-terminal moiety of BteA, but not by the C-terminal moiety of BteA, as expected (Fig 5). These results suggest that the N-terminal moiety of BteA interacts with the C-terminal moiety of BteA and also suggest that an interaction among the C-terminal moieties.

### *B*. *bronchiseptica* internalization by phagocytes

**Fig 6:**
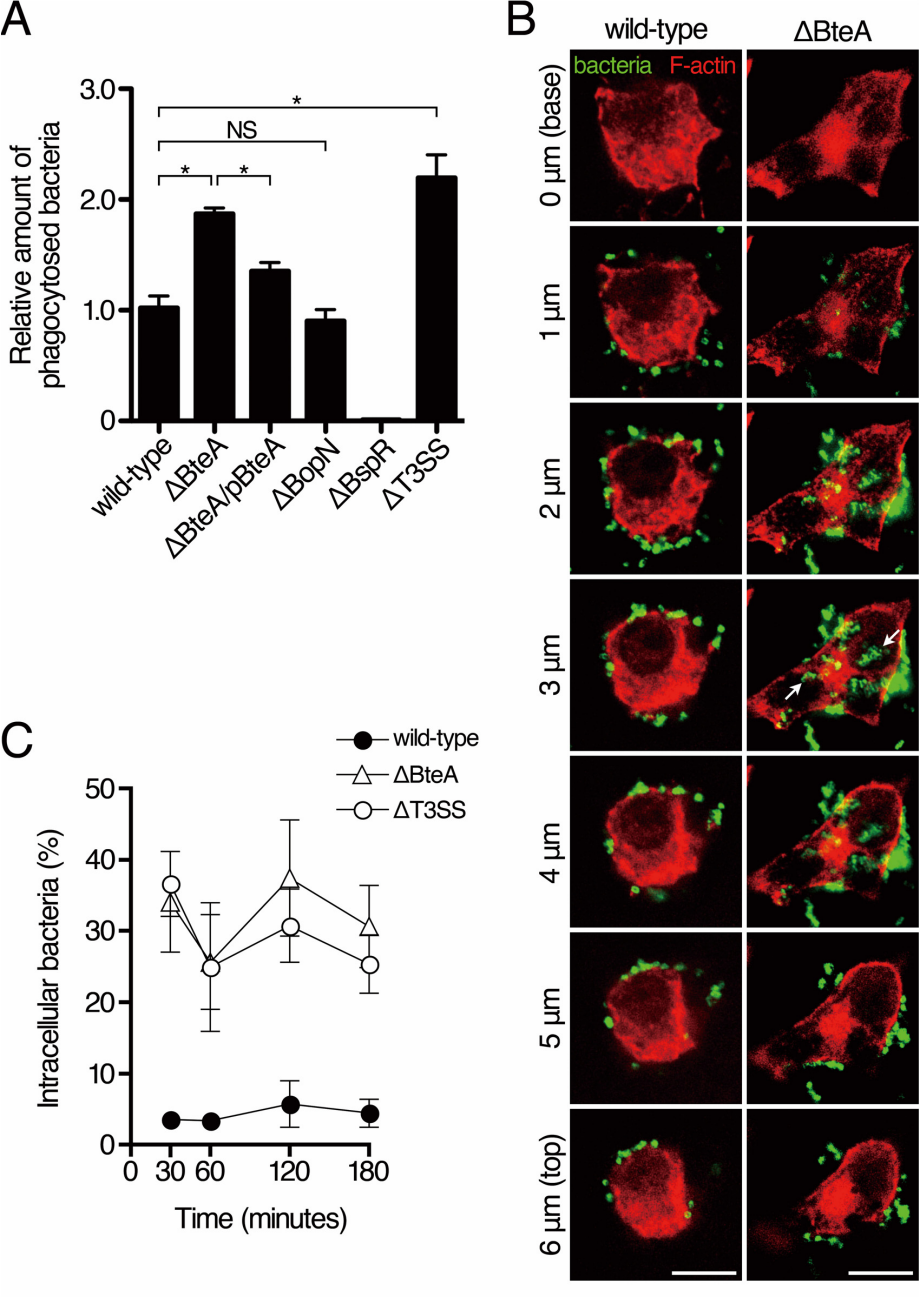
Gentamicin protection assay of J774A.1 cells with *B*. *bronchiseptica* strains. A. J774A.1 cells cultured in 24-well plates were infected with *B*. *bronchiseptica* for 1 hour and extracellular bacteria were then treated with gentamicin for 30 minutes. Phagocytosed bacteria were recovered and plated on LB agar plates in order to grow a single bacterial particle to a visible colony. The number of colonies of each strain was calculated relative to the number of colonies by the wild-type strain infection, which was set as 1.0. Asterisk (*) and NS show a significant difference (*p* < 0.05) and no significant difference, respectively. The error bars represent SEM from triplicate experiments. Representative data from one of three independent experiments were shown. B. Immunofluorescence microscopy of DC2.4 cells infected with the indicated strains for 30 minutes. Infected bacteria (green) and F-actin (red) were stained with anti-*B*. *bronchiseptica* serum and rhodamine phalloidin, respectively. Arrows indicate intracellular bacteria. Scale bar shows 10 μm. Data are representative of three independent experiments. C. The percentage of intracellular bacteria was scored for each of 50 cells. Amounts of intracellular bacteria of ΔBteA or ΔT3SS strain were significantly higher than those of the wild-type strain at each time point (*p* < 0.05). The experiment was performed in tripricate. Representative data from one of three independent experiments were shown.

Phagocytes, such as neutrophils, macrophages, and dendritic cells are recruited to the *Bordetella*-infected respiratory tract [10]. In order to investigate the effects of type III effectors on phagocytosis, the efficiencies of *B*. *bronchiseptica* internalization by phagocytes were measured by a gentamicin protection assay. Cells of a mouse macrophage-like cell line, J774A.1, were infected with the wild-type, ΔBteA, pBteA-introduced ΔBteA (ΔBteA/pBteA), BopN-deficient (ΔBopN), BspR-deficient (ΔBspR), or T3SS-deficient (ΔT3SS) strain. At 1 hour after infection, cells were treated with gentamicin for 30 minutes. The infected cells were then washed and lysed with PBS containing 0.5% Triton X-100. The bacterial suspensions were plated on an LB agar plate and the colonies on the plate were counted as number of the internalized bacteria. The colony number was approximately 0.01% of the input of the wild-type strain (ca. 1 × 10^3^ colonies on the LB plate out of ca. 1 × 10^7^ bacterial particles as input). As shown in Fig 6A, the colony number from the ΔBteA-infected sample was about twice higher than that from the wild-type strain. The colony numbers of strains that deliver BteA into the host cell cytoplasm, such as the wild-type strain, ΔBteA/pBteA, ΔBopN, and ΔBspR, were significantly lower than those of strains that lost the ability to translocate BteA into the host cell cytoplasm, such as ΔBteA and ΔT3SS. To confirm that *B*. *bronchiseptica* inhibits uptake by phagocytic cells, cells of a mouse dendritic cell line, DC2.4, were infected with the wild-type or ΔBteA strain. *B*. *bronchiseptica* and F-actin in DC2.4 cells were stained, and images of infected cells were scanned at 1 μm intervals. The localizations of infected bacteria were analyzed by deconvolution software (Fig 6B). We detected phagocytosed bacteria in DC2.4 cells infected with ΔBteA, whereas in the majority of cells infected with the wild-type strain, phagocytosed bacteria were limited to extracellular surfaces (Fig 6B). In addition, there were significantly fewer intracellular bacteria in DC2.4 cells infected with the wild-type strain than in those infected with ΔBteA or ΔT3SS (Fig 6C). These results suggest that BteA translocated into the host cell cytoplasm was involved in the inhibition of phagocytosis.

## Discussion

As reported previously, morphological changes of L2 cells infected with the wild-type *B*. *bronchiseptica* are induced within 20 minutes after infection [7]. In this conventional protocol, *Bordetella* was in close contact with L2 cells during centrifugation, and indeed, most of the cells had already started to change their morphology in the first time-lapse microscopic image. In order to resolve this problem, we omitted the centrifugation from this experiment. Instead, the multiplicity of infection (MOI) was increased to 1000 from 100 to induce morphological changes efficiently; after this adjustment, we were able to successfully analyze the morphologies of cells infected with *B*. *bronchiseptica* (Fig 1).

The inhibition of necrosis by cytochalasin D (Fig 2) strongly suggests that BteA induces necrosis through the activation or inhibition of a host signaling pathway, rather than via any intrinsic pore-forming activity. There was a possibility that the effector translocation ability of T3SS was inhibited by the addition of cytochalasin D. In order to exclude this possibility, we performed a T3SS-mediated hemolytic assay of *B*. *bronchiseptica* with or without cytochalasin D treatment. We detected no significant difference in hemolytic abilities under either condition (data not shown), indicating that cytochalasin D does not inhibit the T3SS activity. There have been two reports describing the induction of necrotic cell death through actin polymerization. In those studies, *Shigella flexneri*, which is a causative agent of dysentery, was shown to induce both apoptosis and necrosis in a human macrophage cell line and in neutrophils in a T3SS-dependent manner [19, 20]. The neutrophil necrosis induced by the *Shigella* infection is inhibited by cytochalasin D treatment before bacterial infection in vitro, although it is still unknown which *Shigella* type III effectors and host signaling molecules are essential for induction of the necrosis [19]. One report described that mycophenolic acid, which is an immunosuppressor, induces necrosis in activated lymphocytes [21]. The necrosis induction by mycophenolic acid is inhibited by cytochalasin D and requires activation of Cdc42, which is one of the Rho family small GTPases [21]. *B*. *bronchiseptica* induces membrane ruffles at the cell periphery in a BteA-dependent manner (Fig 1). Many reports have described Rac1, a different Rho family small GTPase, as a key molecule for membrane ruffle and lamelipodia formation [18, 22]. In order to examine whether *B*. *bronchiseptica*-induced membrane ruffle depends on Rac1 activities, we performed an infection assay of L2 cells treated with NSC23766, a Rac1 inhibitor. Interestingly, membrane ruffling was induced in the presence of NSC23766 (S1 Fig and S4 Movie). NSC23766 also did not inhibit the LDH release or the detachment of L2 cells infected with the wild-type *B*. *bronchiseptica* (S1 Fig). These results show the possibility that membrane ruffling induced by *B*. *bronchiseptica* does not depend on the Rac1 signaling pathway.

As shown in Figs 3 and 4, the domains responsible for multimerization of BteA (PM domain) and for cytotoxicity (CR domain) were narrowed down to the amino acid region 313–490 and the two regions 200–313 and 400–658, respectively. Although it remains to be elucidated if multimerization of BteA is necessary for necrosis induction, our results show that the N-terminal 199 amino acid region is not essential for the necrosis induction. These results are consistent with the previous report that the N-terminal 130 amino acid region containing the lipid raft targeting (LRT) motif is dispensable for necrosis induction [13]. Although it remains unknown why the separate N- and C-terminal moieties of BteA are able to induce necrosis, it might be interesting to examine whether an interaction between the N- and C-terminal moieties of BteA is necessary for necrosis induction by isolating a mutated BteA lacking the ability for this particular intra/intermolecular interaction. Because of the difficulty of recovering BteA in the lysate of COS-7 cells (Fig 3B), the relationship between the amount of released LDH and the production of BteA proteins in COS-7 cells is unknown. However, western blot analysis did not detect even a slight amount of the full length or C200 of BteA (Fig 3B), suggesting that an undetectable amount of BteA is enough to induce necrosis and that the amount of LDH release might depend on transfection efficiencies. It has been reported that the full length of BteA forms multimer. Because of no signals for C200 of BteA in western blot analysis (Fig 3B), we were unable to determine whether C200 forms multimer or not. On the other hand, N490 (1–490 aa of BteA) forms a multimer, whereas it does not induce LDH release (Fig 3). The introduction of both N400 (1–400 aa of BteA) and C400 (400–658 aa of BteA) into COS-7 cells induces LDH release without multimerization (Fig 4). These results suggest that multimerization of BteA is unnecessary for necrosis induction.

The N-terminal moiety of BteA was precipitated by the C-terminal moiety of BteA in Fig 5. It is unclear why most of the N-terminal moiety remained in the supernatant fraction. However, the interaction among C-terminal moieties might inhibit the interaction between the N- and the C-terminal moieties in our experiment. Identification of the detailed domains will provide characteristics of the BteA conformation in a future work.

ExoU is one of the type III effectors secreted from *Pseudomonas aeruginosa* and induces necrosis when ExoU is produced in cultured mammalian cells by a eukaryotic expression vector [23]. It has been described that ExoU exhibits an activity of phospholipase A2. This report led us to examine whether BteA also has phospholipase A2 activities. However, we were unable to detect any phospholipase A2 activities in the culture supernatant fraction on the *B*. *bronchiseptica* wild-type strain (data not shown) using an EnzChek Phospholipase A2 Assay Kit (Invitrogen). BteA has none of the typical motifs of phospholipase A2, such as a glycine-rich nucleotide binding loop motif (G-X-G-X-X-G/A), serine hydrolase motif (G-X-S-X-G/S), or active site aspartate-containing motif (D-X-G/A), suggesting that BteA induces necrosis through unknown mechanisms that are distinct from the phospholipase A2 pathway exploited by *P*. *aeruginosa* ExoU.

Our previous study demonstrated that the bronchial epithelium of mice infected with wild-type *B*. *bronchiseptica* is not disturbed [10]. This result suggested that BteA might not induce necrosis to epithelial cells in vivo and led us to hypothesize that BteA contributes to alteration of phagocytes that come into contact with *B*. *bronchiseptica* on the respiratory tract during infection. If *B*. *bronchiseptica* induced necrosis in our gentamicin protection assay, the phagocytosed bacteria would also be exposed to the extracellular medium containing gentamicin due to plasma membrane damage during necrosis. The exposed bacteria would be killed by gentamicin and unable to form colonies on an agar plate. In order to avoid *B*. *bronchiseptica*-induced necrosis in this assay, we optimized the assay conditions, removed the centrifugation step, and lowered the MOI. Before we lysed the infected cells in 24-well plates, we removed a portion of the extracellular medium and examined it to ensure that it did not contain a significant amount of LDH. We also optimized the minimum concentration of gentamicin and shortened the duration of gentamicin treatment required to kill *B*. *bronchiseptica* completely. The minimum concentration (1 mg/mL) of gentamicin required to kill *B*. *bronchiseptica* was much higher than the concentration (100 μg/mL) needed to kill *Enterobacteriaceae* such as *E*. *coli* or *Shigella*. The number of detected colonies of the BspR-deficient strain (ΔBspR) was much lower than that of the wild-type strain in the gentamicin protection assay (Fig 6). ΔBspR produces and secretes a greater amounts of BteA than the wild-type strain, and therefore, ΔBspR might inhibit macrophage phagocytosis more strongly than the wild-type strain. In order to examine whether the bactericidal effect of the macrophage degradation pathways contributed to the difference in the phagocytosed bacterial amount between the wild-type and ΔBteA strains, we added bafilomycin A1, a lysosome inhibitor, at 40 or 100 nM as the final concentration with gentamicin. The addition of bafilomycin A1 did not affect the result of the gentamicin protection assay, suggesting that the degradation pathways of macrophages can be excluded in this study.

The relationship between necrosis induction and phagocytosis inhibition is unknown. It might be interesting to investigate whether BteA functions to inhibit phagocytosis even in vivo by animal tests. The molecular mechanism of actin polymerization-dependent necrosis induction also largely remains to be elucidated. In future studies, host counter partner molecules for BteA should be identified to uncover the signal transduction pathways involved in necrosis induction by BteA.

## Supporting Information

S1 Fig *B*. *bronchiseptica* infection with Rac 1 inhibitor, NSC23766, treatment. A. L2 cells were infected with the wild-type *B*. *bronchiseptica* after 1 hour of NSC23766 (Sigma-Aldrich) treatment at 100 μM (+). At 1 hour post-infection, the amount of LDH released into the extracellular medium was measured. The error bars represent the standard error of the mean (SEM) from triplicate experiments. NS shows no significant difference. Representative data from one of three independent experiments. B. NSC23766-treated L2 cells were fixed after 1 hour of infection with the wild-type *B*. *bronchiseptica*. The infected bacteria (green) and F-actin (red) were stained with anti-*B*. *bronchiseptica* antisera and rhodamine phalloidin, respectively. The objectives of 10x (upper photo) and 63x (lower photo) were used to acquire the images. Scale bars in photos obtained with 10x or 63x objective show 100 or 10μm, respectively. Data are representative of three independent experiments. C. Live imaging of a representative L2 cell infected with the wild-type *B*. *bronchiseptica* with NSC23766 treatment. The arrow and arrowhead indicate the membrane ruffling and bleb, respectively. The scale bar shows 10 μm.
(EPS)

S2 Fig Gentamicin protection assay of J774A.1 cells with *B*. *bronchiseptica* with bafilomycin A1 treatment. A. J774A.1 cells cultured in 24-well plates were infected with *B*. *bronchiseptica*. At 1 hour post-infection, extracellular bacteria were then treated with gentamicin at 1 mg/ml and bafilomycin A1 (Sigma-Aldrich) at 40 or 100 nM for 30 minutes. Phagocytosed bacteria were recovered and plated on LB agar plates. The number of colonies was calculated relative to the number of colonies by the wild-type strain infection, which was set as 1.0. Asterisk (*) shows a significant difference (*P* < 0.05).
(EPS)

S1 Movie A representative morphology of the L2 cells infected with the wild-type *B*. *bronchiseptica*. Photos were taken every 1 minute from 5 minutes after infection. The movie is 180 times faster than real time.
(AVI)

S2 Movie A representative morphology of the L2 cell infected with the *B*. *bronchiseptica* ΔBteA. Photos were taken every 1 minute from 5 minutes after infection. The movie is 180 times faster than real time.
(AVI)

S3 Movie A representative morphology of the uninfected L2 cell. Photos were taken every 1 minute from 5 minutes after infection. The movie is 180 times faster than real time.
(AVI)

S4 Movie A representative morphology of the L2 cell infected with the wild-type *B*. *bronchiseptica* with NSC23766 treatment. Photos were taken every 1 minute from 5 minutes after infection. The movie is 180 times faster than real time.
(AVI)

## References

[1] Mattoo S, Cherry JD. Molecular pathogenesis, epidemiology, and clinical manifestations of respiratory infections due to *Bordetella pertussis* and other *Bordetella* subspecies. Clin Microbiol Rev. 2005;18(2):326–82. 10.1128/CMR.18.2.326-382.2005 15831828 PMC1082800

[2] Tang YW, Hopkins MK, Kolbert CP, Hartley PA, Severance PJ, Persing DH. *Bordetella holmesii*-like organisms associated with septicemia, endocarditis, and respiratory failure. Clin Infect Dis. 1998;26(2):389–92. . 9502460 10.1086/516323

[3] Woolfrey BF, Moody JA. Human infections associated with *Bordetella bronchiseptica*. Clin Microbiol Rev. 1991;4(3):243–55. 1889042 10.1128/cmr.4.3.243 PMC358197

[4] Weiss AA, Falkow S. Genetic analysis of phase change in *Bordetella pertussis*. Infect Immun. 1984;43(1):263–9. 6317569 10.1128/iai.43.1.263-269.1984 PMC263420

[5] Yuk MH, Harvill ET, Miller JF. The BvgAS virulence control system regulates type III secretion in *Bordetella bronchiseptica*. Mol Microbiol. 1998;28(5):945–59. . 9663681 10.1046/j.1365-2958.1998.00850.x

[6] Radics J, Konigsmaier L, Marlovits TC. Structure of a pathogenic type 3 secretion system in action. Nat Struct Mol Biol. 2014;21(1):82–7. 10.1038/nsmb.2722 . 24317488

[7] Kuwae A, Ohishi M, Watanabe M, Nagai M, Abe A. BopB is a type III secreted protein in *Bordetella bronchiseptica* and is required for cytotoxicity against cultured mammalian cells. Cell Microbiol. 2003;5(12):973–83. . 14641181 10.1046/j.1462-5822.2003.00341.x

[8] Nogawa H, Kuwae A, Matsuzawa T, Abe A. The type III secreted protein BopD in *Bordetella bronchiseptica* is complexed with BopB for pore formation on the host plasma membrane. J Bacteriol. 2004;186(12):3806–13. 10.1128/JB.186.12.3806-3813.2004 15175294 PMC419950

[9] Bibova I, Hot D, Keidel K, Amman F, Slupek S, Cerny O, et al Transcriptional profiling of *Bordetella pertussis* reveals requirement of RNA chaperone Hfq for Type III secretion system functionality. RNA Biol. 2015;12(2):175–85. 10.1080/15476286.2015.1017237 . 25674816 PMC4615762

[10] Nagamatsu K, Kuwae A, Konaka T, Nagai S, Yoshida S, Eguchi M, et al *Bordetella* evades the host immune system by inducing IL-10 through a type III effector, BopN. J Exp Med. 2009;206(13):3073–88. 10.1084/jem.20090494 20008527 PMC2806459

[11] Panina EM, Mattoo S, Griffith N, Kozak NA, Yuk MH, Miller JF. A genome-wide screen identifies a *Bordetella* type III secretion effector and candidate effectors in other species. Mol Microbiol. 2005;58(1):267–79. 10.1111/j.1365-2958.2005.04823.x . 16164564

[12] Kuwae A, Matsuzawa T, Ishikawa N, Abe H, Nonaka T, Fukuda H, et al BopC is a novel type III effector secreted by *Bordetella bronchiseptica* and has a critical role in type III-dependent necrotic cell death. J Biol Chem. 2006;281(10):6589–600. 10.1074/jbc.M512711200 . 16407269

[13] French CT, Panina EM, Yeh SH, Griffith N, Arambula DG, Miller JF. The *Bordetella* type III secretion system effector BteA contains a conserved N-terminal motif that guides bacterial virulence factors to lipid rafts. Cell Microbiol. 2009;11(12):1735–49. 10.1111/j.1462-5822.2009.01361.x 19650828 PMC2788067

[14] Kurushima J, Kuwae A, Abe A. The type III secreted protein BspR regulates the virulence genes in *Bordetella bronchiseptica*. PLoS One. 2012;7(6):e38925 10.1371/journal.pone.0038925 22701731 PMC3372540

[15] Stainer DW, Scholte MJ. A simple chemically defined medium for the production of phase I *Bordetella pertussis*. J Gen Microbiol. 1970;63(2):211–20. . 4324651 10.1099/00221287-63-2-211

[16] Schneider CA, Rasband WS, Eliceiri KW. NIH Image to ImageJ: 25 years of image analysis. Nat Methods. 2012;9(7):671–5. . 22930834 10.1038/nmeth.2089 PMC5554542

[17] Kuwae A, Yoshida S, Tamano K, Mimuro H, Suzuki T, Sasakawa C. *Shigella* invasion of macrophage requires the insertion of IpaC into the host plasma membrane. Functional analysis of IpaC. J Biol Chem. 2001;276(34):32230–9. 10.1074/jbc.M103831200 . 11413141

[18] Hall A. Rho GTPases and the actin cytoskeleton. Science. 1998;279(5350):509–14. . 9438836 10.1126/science.279.5350.509

[19] Francois M, Le Cabec V, Dupont MA, Sansonetti PJ, Maridonneau-Parini I. Induction of necrosis in human neutrophils by *Shigella flexneri* requires type III secretion, IpaB and IpaC invasins, and actin polymerization. Infect Immun. 2000;68(3):1289–96. 10678940 10.1128/iai.68.3.1289-1296.2000 PMC97281

[20] Nonaka T, Kuwabara T, Mimuro H, Kuwae A, Imajoh-Ohmi S. *Shigella*-induced necrosis and apoptosis of U937 cells and J774 macrophages. Microbiology. 2003;149(Pt 9):2513–27. . 12949176 10.1099/mic.0.26341-0

[21] Chaigne-Delalande B, Guidicelli G, Couzi L, Merville P, Mahfouf W, Bouchet S, et al The immunosuppressor mycophenolic acid kills activated lymphocytes by inducing a nonclassical actin-dependent necrotic signal. J Immunol. 2008;181(11):7630–8. . 19017951 10.4049/jimmunol.181.11.7630

[22] Ridley AJ, Paterson HF, Johnston CL, Diekmann D, Hall A. The small GTP-binding protein rac regulates growth factor-induced membrane ruffling. Cell. 1992;70(3):401–10. . 1643658 10.1016/0092-8674(92)90164-8

[23] Sato H, Frank DW, Hillard CJ, Feix JB, Pankhaniya RR, Moriyama K, et al The mechanism of action of the Pseudomonas aeruginosa-encoded type III cytotoxin, ExoU. EMBO J. 2003;22(12):2959–69. 10.1093/emboj/cdg290 12805211 PMC162142

