# Correction: BteA Secreted from the *Bordetella bronchiseptica* Type III Secetion System Induces Necrosis through an Actin Cytoskeleton Signaling Pathway and Inhibits Phagocytosis by Macrophages

**DOI:** 10.1371/journal.pone.0180287

**Published:** 2017-06-22

**Authors:** Asaomi Kuwae, Fumitaka Momose, Kanna Nagamatsu, Yasuharu Suyama, Akio Abe

There are multiple spelling errors in this article. The word “secretion” is spelled incorrectly. The correct title is: BteA Secreted from the *Bordetella bronchiseptica* Type III Secretion System Induces Necrosis through an Actin Cytoskeleton Signaling Pathway and Inhibits Phagocytosis by Macrophages.

There is an error in the current address for the third author. The address should read: Department of Molecular Microbiology, Washington University, Saint Louis, MO 63110, United States of America.

There is an error in the eleventh sentence in the second paragraph of the Discussion section. The sentence should read: Many reports have described Rac1, a different Rho family small GTPase, as a key molecule for membrane ruffle and lamellipodia formation [18, 22].
